# Treosulfan Exposure Predicts Thalassemia-Free Survival in Patients with Beta Thalassemia Major Undergoing Allogeneic Hematopoietic Cell Transplantation

**DOI:** 10.1002/cpt.3078

**Published:** 2023-11-07

**Authors:** Aswin Anand Pai, Ezhilpavai Mohanan, John C. Panetta, Uday P. Kulkarni, Raveen Stephen Stallon Illangeswaran, Balaji Balakrishnan, Agila Jayaraman, Eunice S. Edison, Kavitha M. Lakshmi, Anup J. Devasia, Nambiathayil Aboobacker Fouzia, Anu Korula, Aby Abraham, Biju George, Alok Srivastava, Vikram Mathews, Joseph F. Standing, Poonkuzhali Balasubramanian

**Affiliations:** 1 Department of Hematology, Christian Medical College, Vellore, India; 2 Sree Chitra Tirunal Institute for Medical Sciences and Technology, Thiruvananthapuram, India; 3 Department of Pharmaceutical Sciences, St Jude Children’s Research Hospital, Memphis, Tennessee, USA; 4 Infection, Immunity and Inflammation, Great Ormond Street Institute of Child Health, University College London, London, UK; 5 Department of Pharmacy, Great Ormond Street Hospital for Children, NHS Foundation Trust, London, UK

## Abstract

A toxicity-reduced conditioning regimen with treosulfan, fludarabine, and thiotepa in patients with high-risk β-thalassemia major has significantly improved hematopoietic stem cell transplantation (HCT) outcomes. However, complications resulting from regimen-related toxicities (RRTs), mixed chimerism, and graft rejection remain a challenge. We evaluated the dose–exposure-response relationship of treosulfan and its active metabolite S, S-EBDM, in a uniform cohort of patients with β-thalassemia major to identify whether therapeutic drug monitoring (TDM) and dose adjustment of treosulfan is feasible. Plasma treosulfan/S, S-EBDM levels were measured in 77 patients using a validated liquid chromatography with tandem mass spectrometry method, and the pharmacokinetic parameters were estimated using nlmixr2. The influence of treosulfan and S, S-EBDM exposure, and *GSTA1/NQO1* polymorphisms on graft rejection, RRTs, chimerism status, and 1-year overall survival (OS), and thalassemia-free survival (TFS) were assessed. We observed that treosulfan exposure was lower in patients with graft rejection than those without (1,655 vs. 2,037 mg•h/L, *P* = 0.07). Pharmacodynamic modeling analysis to identify therapeutic cutoff revealed that treosulfan exposure ≥1,660 mg•hour/L was significantly associated with better 1-year TFS (97% vs. 81%, *P* = 0.02) and a trend to better 1-year OS (90% vs. 69%, *P* = 0.07). Further, multivariate analysis adjusting for known pre-HCT risk factors also revealed treosulfan exposure <1,660 mg•h/L (hazard ratio (HR) = 3.23; 95% confidence interval (CI) = 1.12–9.34; *P* = 0.03) and GSTA1*B variant genotype (HR = 3.75; 95% CI = 1.04–13.47; *P* = 0.04) to be independent predictors for inferior 1-year TFS. We conclude that lower treosulfan exposure increases the risk of graft rejection and early transplant-related mortality affecting TFS. As no RRTs were observed with increasing treosulfan exposure, TDM-based dose adjustment could be feasible and beneficial.

Treosulfan has been increasingly used as part of the conditioning regimen for allogeneic hematopoietic stem cell transplantation (HCT) recipients with hematological malignancies^1–4^ and benign conditions.^5,6^ Treosulfan, administered as a prodrug, is converted nonenzymatically and at physiological temperature and pH 7.42 to its active metabolites monoepoxide (2S, 3S)-1,2-epoxy butane-3,4-diol-4-methanesulfonate (S, S-EBDM) and (2S,3S)-1,2:3,4-diepoxybutane (S, S-DEB), the latter of which causes cytotoxicity.^7^ Since treosulfan metabolism takes place nonenzymatically, there was no previous study that explored the possibility of genetic polymorphisms in the genes encoding enzymes in their metabolic pathway. As treosulfan is structurally very similar to busulfan and the downstream active metabolites undergo similar mechanism of action by alkylating the guanine bases, we attempted to look at any genetic factors that influence further downstream activity, especially the DNA–DNA or DNA–protein crosslinking, which is essential for the cell kill. In an unpublished ongoing work,^8^ we explored the drug-metabolizing enzymes and transporters (DMET) genes associated with fludarabine metabolism in the fludarabine-treosulfan combination regimen, and, surprisingly, we identified polymorphisms in *GSTA4, NQO1, GSTZ1*, and *CES2* genes to be significantly associated with treosulfan pharmacokinetics (PK). The mechanism by which these polymorphisms influence treosulfan PK is yet to be identified in the HCT setting.

HCT remains the only curative modality for patients with β-thalassemia major (TM).^9–11^ The introduction of a toxicity-reduced conditioning regimen containing treosulfan, fludarabine, and thiotepa and a peripheral blood stem cell graft in patients with class III TM has significantly improved transplant outcomes, with a reduction in early transplant-related mortality (TRM) from 46% (in historical busulfan/cyclophosphamide regimen) to 13%.^12–14^ However, complications related to mixed chimerism (MC), graft rejection, and regimen-related toxicity (RRTs) still pose a significant concern, limiting the success of HCT,^15,16^ suggesting an unmet need for further optimizing the treosulfan-based regimen.

Recent dose-exposure-response studies suggest significant interindividual variation (IIV) in treosulfan exposure resulting in variable HCT outcomes, reinforcing the importance of therapeutic drug monitoring (TDM) of treosulfan.^17^ However, the therapeutic range of treosulfan is not clearly defined due to conflicting data.^16–18^ Recent evidence also suggests that S, S-EBDM plays a more significant role than treosulfan and S, S-DEB in mediating cytotoxic effects.^19^ However, there is no data on metabolite S, S-EBDM influencing HCT outcomes. Here, we describe the PK, pharmacogenetics, and pharmacodynamics (PD) of treosulfan and its metabolite S, S-EBDM in patients with TM. We also aimed to validate the impact of our previously reported genetic polymorphisms in the present study cohort.

## Patients and Methods

### Patients

Patients with TM receiving a treosulfan/fludarabine/thiotepa regimen before HCT between March 2017 and April 2021 were included in the study after obtaining written informed assent or consent from the parents/patients, respectively. The institutional review board approved this study (IRB No: 9411, dated April 29, 2015). The patients were risk-stratified based on Vellore risk classification as reported previously.^12^

### Conditioning regimen and GVHD prophylaxis

With the aim of reducing TRM and graft rejection in high-risk patients with TM, our center has been utilizing the treosulfan/fludarabine/thiotepa-based toxicity-reduced conditioning regimen since 2009.^13^ All patients received fludarabine at a dose of 40 mg/m^2^/day as a 1-hour infusion from Day −5 to Day −2 and treosulfan at 14 g/m^2^/day at the rate of 5 g/hour from Day −5 to Day −3 and a single dose of thiotepa on Day −6 before HCT. Cyclosporin and a short course of methotrexate were used as graft-vs.-host disease (GVHD) prophylaxis.

### Chimerism analysis

Whole blood chimerism was analyzed post-HCT using polymerase chain reaction to amplify the informative short tandem repeat or variable number tandem repeat in recipient and donor samples, followed by gene scan analysis as previously reported.^20^

### Reagents and chemicals for treosulfan and metabolite measurement

Treosulfan used for preparing both treosulfan and S, S-EBDM standards was a kind gift from Medac (Hamburg, Germany). Compounds 4′-aminoacetophenone (internal standard), formic acid, ammonium formate, and citric acid were obtained from Sigma–Aldrich (St Louis, MO). Sodium hydroxide was purchased from Merck KGaA (Darmstadt, Germany). Acetonitrile and LC/MS grade methanol were purchased from Fisher Scientific (Thermo Fisher Scientific, Waltham, MA). High-performance liquid chromatography (HPLC)–grade water (Qualigens, India) was used as the solvent. The treosulfan/S, S-EBDM assay standards were prepared in drug-free blank plasma (Christian Medical College hospital blood bank).

### Blood sample collection and processing

Heparinized peripheral blood samples were collected just before (0 hour) and at the end of infusion and 2, 4, and 24 hours after treosulfan infusion based on the limited sampling model reported previously by us.^21^ Blood samples were immediately adjusted to a pH of 5.5 by adding 50 μL of 1 M citric acid/mL of blood to avoid the artificial *ex vivo* conversion of treosulfan to S, S-EBDM. The sample was centrifuged at 13,000 rpm for 5 minutes to obtain plasma, which was stored at –80°C until further analysis. Sample processing for liquid chromatography with tandem mass spectrometry (LC–MS/MS) assay was carried out as reported previously with a few modifications.^22^ Briefly, stock solutions containing both treosulfan and S, S-EBDM were prepared by alkalinization with 1 N sodium hydroxide solution, following which the calibration standards were prepared by serial dilution in HPLC-grade water. Then, 50 μL of the acidified plasma was spiked with 50 μL of each standard solution/water for patient samples and 50 μL of IS, vortexed, precipitated with 150 μL of 100% acetonitrile, and centrifuged at 13,000 rpm at 4°C for 5 minutes. The resulting clear filtrate (1 μL) was injected via an autosampler for analysis in LC–MS/MS.

### Quantification of treosulfan and metabolite, S, S-EBDM in plasma

Treosulfan and S, S-EBDM levels in plasma were measured using a Shimadzu-Nexera X2 ultra HPLC coupled with an LCMS-8050 triple quadrupole mass spectrometer (Shimadzu, Kyoto, Japan). The parameters were adjusted to yield maximum multiple reaction monitoring signals (Table S1). Chromatographic separation of the analytes was done using Zorbax Eclipse Plus C18 (100 mm × 2.1 mm, 3.5 μm; Agilent, CA) protected with a C18 guard column from the same source using isocratic elution with a mobile phase 0.01 M ammonium formate buffer at a flow rate of 0.4 mL/minute maintained at 40°C. The total run time was 5.5 minutes (Figure S1). The chromatograms were analyzed using LC Solutions software (Shimadzu, Kyoto, Japan).

### Pharmacogenetic evaluation

We had previously screened for all genetic variants using DMET Plus Premier Pack (Affymetrix, Santa Clara, CA, USA) in 51 patients with TM who received the treosulfan-fludarabine-thiotepa regimen. The effect of genetic polymorphisms (at an allele frequency of >0.05%) on treosulfan PK was evaluated (S, S-EBDM PK was not assessed then). We identified four polymorphisms that were significantly associated with treosulfan PK-3′UTR variants in *GSTA4* (glutathione S-transferase; rs7496) and *NQO1* (NAD(P)H dehydrogenase 1; rs10517), a missense variant in *GSTZ1* (rs1046428), and an intronic variant in *CES2* (carboxylesterase 2; rs4783745) genes.

In the present study, the patient’s preconditioning peripheral blood was collected in EDTA tubes, and DNA was extracted using the Gentra Puregene kit (QIAGEN, Hilden, Germany). We screened for the above four polymorphisms with GSTA1*B (glutathione S-transferase haplotype comprising promoter polymorphisms: rs3957356 and rs3957357) using a genotyping array (Standard BioTools, CA, USA).

### Population pharmacokinetics (PopPK) of treosulfan and S, S-EBDM

We tested one-compartment and two-compartment models to explain both compounds’ metabolism, including additive and proportional residual errors. Allometric weight scaling for clearance and volume was tested. Covariates such as age and serum creatinine were tested on clearance. The typical body weight of the population was set to 70 kg, and the exponential term was set as 0.75 for clearance and 1 for volume. The addition of covariates was considered if the −2 log-likelihood ratio significantly improved the fit at the least by *P* < 0.01 of the model. Further, the model was evaluated by a series of the goodness-of-fit predicted vs. observed plots, visual predictive check plots, residual vs. time, and prediction plots.

### End points and definitions

HCT outcome data such as RRTs, engraftment, graft rejection, GVHD, post-HCT chimerism, and survival status were collected through a retrospective chart review for at least 1 year post-HCT. An absolute neutrophil count of ≥500 × 10^6^/L on three consecutive days was defined as neutrophil engraftment; Day +28 chimerism analysis showing more than 95% of donor genetic marker patterns was considered as achieving complete chimerism. MC was defined as the presence of >5% residual host chimerism at any time post-HCT, rejection as >90% residual host chimerism in peripheral blood, as described previously.^23^ The RRTs, including mucositis, were graded according to NCI-CTCAE criteria.^24^ Hepatic sinusoidal obstruction syndrome (SOS) was graded according to Baltimore criteria.^25^ GVHD was graded using Glucksberg criteria.^25^ Any deaths occurring within the first 100 days post-HCT were regarded as TRM. Early TRM (TRM Day +30) and late TRM (TRM Day +100) are deaths occurring within 30 and 100 days post-HCT, respectively. One-year thalassemia-free survival (TFS) was defined from the time of transplant to an event; an event was primary graft rejection/failure or death up to 1 year post-HCT. The 1-year OS was defined as the percentage of patients alive at the last follow-up at 1 year post-HCT.

### Statistical analyses

The patient’s pre-HCT characteristics were summarized by standard descriptive statistics. Individual treosulfan, S, S-EBDM exposure, and parent-to-metabolite exposure ratio (S, S-EBDM AUC/treosulfan AUC) were continuous variables for the outcome analysis. The correlation between treosulfan and S, S-EBDM exposure was estimated using Pearsons coefficient correlation. Associations between treosulfan and S, S-EBDM exposure, genotype groups, and clinical outcomes were done using Mann–Whitney U-test. Multivariate Cox proportional hazards regression models were developed to identify the factors associated with time to 1-year TFS. Kaplan–Meier survival analysis was used to estimate the 1-year TFS and OS probabilities. All statistical analyses were performed by R Statistical software (version 4.3.0; R Foundation for Statistical Computing, Vienna, Austria) and GraphPad Prism software (version 8.4.3; GraphPad Software Inc, San Diego, CA, USA).

### Pharmacodynamic modeling

A Cox proportional hazard analysis was performed to determine the factors associated with the outcomes of post-HCT, especially graft rejection and TFS. The covariates included were treosulfan area under the curve (AUC), S, S-EBDM AUC, treosulfan/S, S-EBDM AUC ratio, and genetic polymorphisms, in addition to the standard demographic variables such as age, sex, Lucarelli classification, CD34 dose, donor source, and human leukocyte antigen (HLA) match on time to rejection and time to death at the end of 1 year. Significant variables (*P* < 0.05) from this univariate analysis were taken forward for multivariate analysis. From the median AUC values, we empirically tested the target therapeutic range or cutoff for treosulfan AUC with Kaplan–Meier survival curves for mortality and rejection. A generalized linear model with binomial error distribution was fitted to check the probability of success with the target AUC values (defined as being alive at the last follow-up (1 year post-HCT), with <5% probability of rejection) was fitted. All the analyses were done using R (version 4.3.0, R Foundation for Statistical Computing).

## Results

### Patient demographics

Seventy-seven patients with TM underwent HCT in our center between March 2017 and April 2021 and followed up for at least 1 year post-HCT enrollment. Most patients were in class III (class III high risk: 25%; class III low risk: 53%), and 19% belonged to the class II risk category. Detailed demographics of these patients are summarized in Table 1.

### HCT outcomes

Of the 77 patients, 75 (97.4%) engrafted at a median of 16 days post-HCT (range: 12–43 days), while two did not engraft (2.6%) and subsequently died. Post-HCT chimerism was evaluated in all patients alive beyond Day +28 (*n* = 75). Eight patients (10.4%) had MC on Day +28. Five patients (6.5%) had graft rejection within 1 year, in which four patients subsequently died and one survived post-second HCT. Nineeteen (25%) developed hepatic SOS. Mucositis grades II–IV was observed in 40 (52%) patients; 3 (4%) developed grade I mucositis, and 17 of the 75 evaluable patients developed acute GVHD (23%). The early (D +30) and late (D +100) TRM were 5.2% and 10.4%, respectively, while the 1-year OS and TFS were 83% and 82%, respectively.

### Bioanalytical method to measure treosulfan and S, S-EBDM in plasma

Before analyzing patient plasma samples, we validated the treosulfan and S, S-EBDM bioanalytical method for its specificity, linearity, precision, accuracy, and recovery. The assay was linear for a concentration range of 23–5,720 μM and 17–8,723 μM for treosulfan and S, S-EBDM, respectively, with mean *R*^2^ = 0.99 ± 0.001. The interday assay accuracy and precision were >90% and 95%, respectively (Table S2). The mean recovery of treosulfan and S, S-EBDM from plasma was 95–100%, which signifies that there was no loss of the analyte during analytical processing. There was no matrix interference. The lower limit of quantitation of the assay for both treosulfan and S, S-EBDM were 1.4 and 2.1 μM, respectively, and the limit of detection values were 0.7 and 1.05 μM for treosulfan and S, S-EBDM, respectively.

### Treosulfan and S, S-EBDM PK

**Figure 1:**
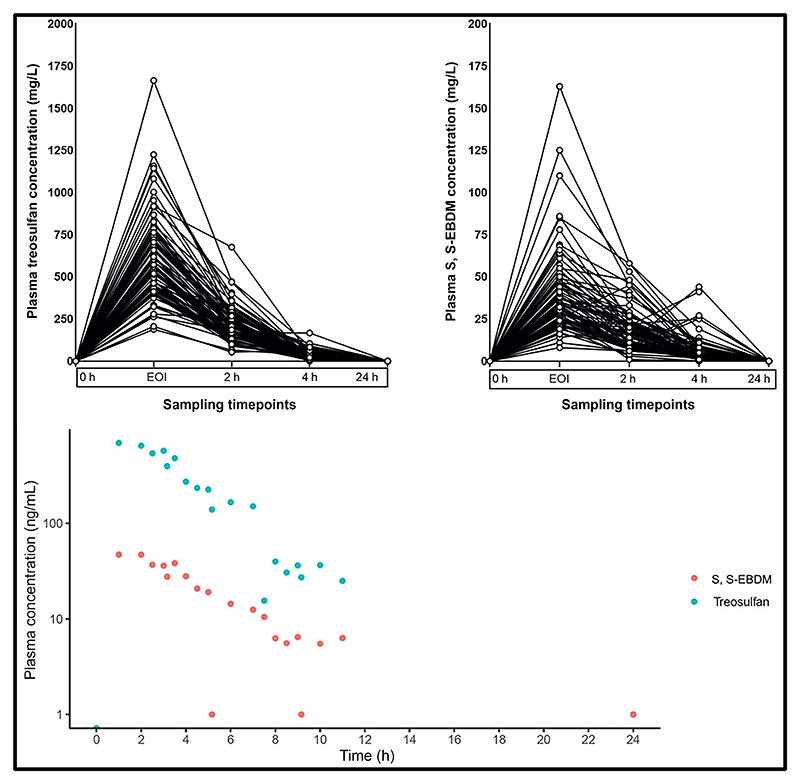
Plots showing treosulfan and S, S-EBDM concentrations vs. timepoints in the study cohort. Spaghetti plots representing (**top left**) treosulfan and (**top right**) S, S-EBDM concentrations vs. timepoints acquired for patients included in the study. Plasma treosulfan and S, S-EBDM levels (mg/L) were plotted against five study timepoints (0-hour pre-treosulfan infusion, EOI (end of treosulfan infusion), and 2, 4, and 24 hours post-treosulfan infusion). Log plasma concentration-time profile (**bottom**) of both treosulfan and S, S-EBDM for patients included in the study. Mean log-transformed treosulfan and S, S-EBDM concentrations were plotted against actual study timepoints.

**Figure 2:**
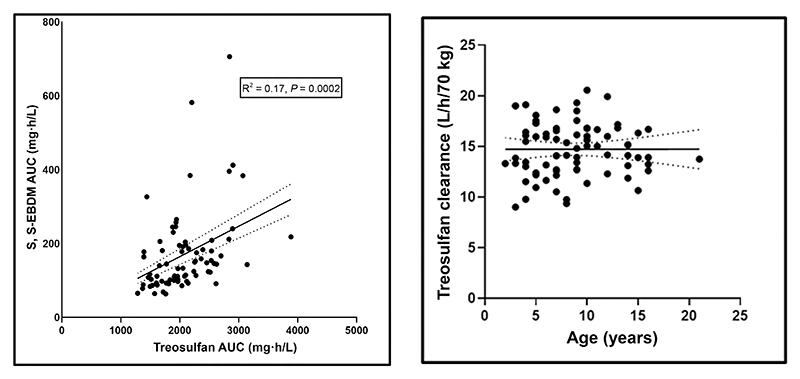
Correlation analysis: (**left**) treosulfan with S, S-EBDM exposure, and (**right**) treosulfan clearance with age. **Left**: correlation analysis between the (x-axis) treosulfan and (y-axis) S, S-EBDM exposure in each patient showed a statistically moderate positive linear relationship (*R*^2^ = 0.17, *P* = 0.0002). **Right**: Correlation analysis between (x-axis) age and (y-axis) treosulfan clearance in each patient showing absence of correlation.

The median *C*_max_ (peak plasma concentration at the end of conditioning) and range of plasma treosulfan and S, S-EBDM levels were 560 (188–1,665) and 33 (8–163) mg/L, respectively. The 24-hour samples had not detected treosulfan or S, S-EBDM levels in all patients. From 77 patients, 385 plasma samples were available to develop the PopPK model for treosulfan and its metabolite. The time-concentration profiles for treosulfan and S, S-EBDM are presented in Figure 1. PopPK analysis was done using nlmxir2 in R (4.3.0). We built a base model with one compartment with an allometric weight scale on clearance and volume, which best described the PK of treosulfan. Postmenstrual calculated age and serum creatinine were tested as covariates on the model, which did not significantly improve the model. Finally, one compartment model with allometric scaling on clearance and volume of distribution and combined additive-proportional residual error for both parent and metabolite were developed and validated using the goodness-of-fit predicted vs. observed plots, visual predictive check plots, residual vs. time, and prediction plots (Figure S2). There was a twofold variability in treosulfan exposure (1,993 (1,286–3,886 mg•h/L)) and 10-fold variability in S, S-EBDM exposure (143 (64–706 mg•h/L)). We also observed a moderate positive linear relationship between treosulfan and S, S-EBDM exposure (*R*^2^ = 0.17, *P* = 0.0002). We did not observe any correlation between age and treosulfan clearance (Figure 2).

### Influence of GSTA1*B and *NQO1* 3′UTR polymorphisms on treosulfan and S, S-EBDM exposure

**Figure 3:**
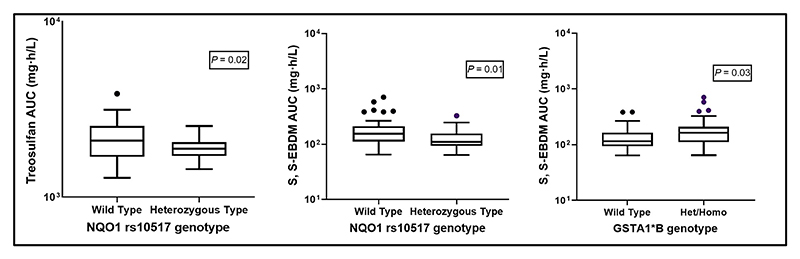
*NQO1* / GSTA1*B genotypes influence treosulfan and S, S-EBDM Exposure. Association between *NQO1* and GSTA1*B genotypes with treosulfan and S, S-EBDM exposure. *Wild-type: homozygous reference genotype; Het/Homo: heterozygous and homozygous genotype; the *P* value was calculated by Mann–Whitney *U*-test. *Asterisks indicate the level of the significance (*P* value); *Means *P* < 0.05. Log-transformed (y-axis) treosulfan and S, S-EBDM AUC were plotted against (x-axis) NQO1 / GSTA1*B genotypes. AUC, area under the concentration-time curve.

Table 2 describes the distribution of wild-type and variant genotypes of the selected polymorphisms in this patient cohort. The allele frequencies did not vary from the Indian population^26^ and all the polymorphisms tested were consistent with Hardy–Weinberg equilibrium. When tested whether the polymorphisms influence treosulfan and metabolite PK, two of the five polymorphisms screened (GSTA1*B and *NQO1*) were significantly associated with treosulfan and/or S, S-EBDM exposure. Treosulfan AUC was significantly lower in patients with variant genotype (*n* = 26) for *NQO1* rs10517 polymorphism compared with those with wild-type (*n* = 51) genotype (1,890 (1,441–2,537 mg•h/L) vs. 2,100 (1,286–388 mg•h/L), *P* = 0.02). S, S-EBDM exposure was significantly higher in patients with variant genotype for GSTA1*B polymorphism (*n* = 41) compared with those with wild-type gen-otype (*n* = 36; 164 (65–706 mg•h/L) vs. 115 (64–385 mg•h/L), *P* = 0.03). S, S-EBDM exposure was significantly lower in patients with variant genotype (*n* = 26) for *NQO1* rs10517 polymorphism compared with those with wild-type genotype (*n* = 51; 110 (64–327 mg•h/L) vs. 154 (65–706 mg•h/L), *P* = 0.01 (Figure 3)).

### Impact of treosulfan and S, S-EBDM exposure on HCT outcomes

We then evaluated the influence of treosulfan AUC and S, S-EBDM exposure on HCT outcome parameters, including engraftment, rejection, RRTs, TRM, OS, and TFS. Treosulfan AUC was lower in patients who had graft rejection vs. those who did not (1,655 vs. 2,037 mg•h/L (*P* = 0.07)), showing a trend to significance. There were no associations between treosulfan or S-S-EBDM AUC and other HCT outcomes, including engraftment or RRTs. Patients who developed SOS had had a significantly low treosulfan/S, S-EBDM AUC ratio compared with patients who did not develop SOS (13 (4.4–20.2) vs. 17 (3.8–28.6), *P* = 0.04). Again, on quartile analysis, we observed that patients with high treosulfan/S, S-EBDM AUC >18.5 had a decreased incidence of SOS (5% vs. 31%, *P* = 0.03), suggesting that increased conversion of treosulfan to S, S-EBDM could increase the incidence of RRTs. None of the other HCT outcomes, including rejection or survival, was influenced by the treosulfan/S, S-EBDM exposure ratio.

### Influence of genetic polymorphisms on HCT outcomes

We then assessed the impact of the screened genetic polymorphisms on early HCT outcomes. While no associations were observed between genotypes and engraftment, chimerism status, or RRTs, we observed that patients with variant genotypes for *NQO1* rs10517 polymorphism had significantly inferior 1-year OS (69.2% vs. 92.2%, *P* = 0.008) and 1-year TFS (69.2% vs. 88.2%, *P* = 0.04 (Figure S3A)) compared with the patients who carried wild-type genotypes. Similarly, patients with variant genotypes for GSTA1*B polymorphism had significantly inferior 1-year OS (75.6% vs. 94.4%, *P* = 0.02) and 1-year TFS (73.2% vs. 91.7%, *P* = 0.04 (Figure S3B)) compared with the patients who carried wild-type genotypes.

### PD modeling and optimal exposure prediction

**Figure 4:**
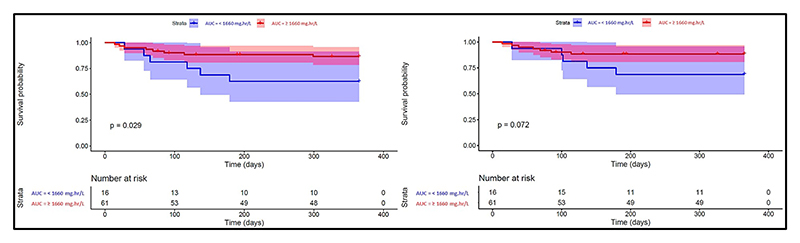
Role of treosulfan AUC in predicting the probability of 1-year TFS and OS. Kaplan–Meier curve for (**left**) 1-year TFS and (**right**) OS in patients above and below the upper success probability treosulfan AUC cutoff of 1,660 mg•hour/L. AUC, area under the concentration-time curve; OS, overall survival; TFS, thalassemia-free survival.

Since we found low treosulfan area under the plasma concentration–time curve from time zero to infinity (AUC_(0–∞)_) to be associated with graft rejection (trend to significance), we then used the PD model to find the most predictive treosulfan exposure measure AUC_(0–∞)_ for better 1-year OS and TFS. The 1-year OS and TFS were modeled stepwise using Cox proportional hazards. There was a trend for low treosulfan AUC_(0–∞)_ to be associated with rejection (hazard ratio (HR) (95% confidence interval (CI)) = 0.08 (0, 1.37); *P* = 0.08); four covariates were significant by association with 1-year mortality (*GSTA1, NQO1* polymorphisms, incidence of acute GVHD and hepatic SOS (Table S3)). We then attempted to find the optimal cutoff for treosulfan exposure that could give the maximum probability of better 1-year TFS and OS. Treosulfan exposure ≥1,660 mg•hour/L was significantly associated with better 1-year TFS (88.5% vs. 62.5%, *P* = 0.029) and trend to better 1-year OS (90.2% vs. 68.8%, *P* = 0.07 (Figure 4). On multivariate Cox regression analysis, only treosulfan AUC <1,660 mg•hour/L (HR = 3.23; 95% CI = 1.12–9.34; *P* = 0.03) and GSTA1*B variant genotype (HR = 3.75; 95% CI = 1.04–13.47; *P* = 0.04) independently predicted inferior 1-year TFS (Table 3).

## Discussion

We established and validated a robust, rapid, and cost-effective LC–MS/MS-based method to quantify treosulfan and S, S-EBDM concentrations simultaneously in patients’ plasma samples. Previously high-performance liquid chromatography with refractive index detection was used to measure treosulfan and its metabolites.^21,27^ However, this method is limited by its low sensitivity and specificity compared with LC–MS/MS. HPLC with ultraviolet detection (HPLC-UV) methods have also been described to measure treosulfan and its metabolites.^28,29^ Due to the time-consuming and laborious derivatization for HPLC-UV assays, LC–MS/MS assays are superior and feasible for routine analysis. Romański *et al*.^22^ established an LC–MS/MS assay to determine treosulfan and S, S-EBDM in plasma and tissue samples. We adopted the same methodology with minor modifications (use of a different internal standard and change in elution protocol). The advantages of the assay, such as the short run-time and the need for minimal plasma samples (50 μL), make it an ideal methodology for TDM purposes.

Treosulfan PK has been reported previously in patients undergoing HCT for malignant and benign conditions and in various combinations.^21,28–33^ PopPK analysis in the present study revealed a twofold and 10-fold variability in treosulfan and S, S-EBDM exposure, respectively. The treosulfan PK estimates were comparable to the previous studies (Table S4). Except for a pilot study,^34^ there are no extensive reports on S, S-EBDM PK to date. The authors observed large IIV (14-fold) in S, S-EBDM exposure similar to ours, but they did not address the impact of the metabolite exposure on HCT outcomes. Despite a similar half-life, S, S-EBDM levels were 2-magnitude lower than treosulfan levels, suggesting rapid elimination as reported previously.^35,36^ Also, there was no strong correlation between treosulfan and S, S-EBDM exposure, suggesting the lack of a linear relationship between treosulfan and S, S-EBDM exposure. Genetic and other physiological factors could explain nonlinearity and high IIV in S, S-EBDM exposure.

Recent dose-exposure-response studies^18,37^ have attempted to derive a therapeutic range for treosulfan exposure for improved HCT outcomes. Our previous study^21^ evaluated treosulfan PK in 87 patients with β-thalassemia major, but we failed to observe clear-cut clinical associations with treosulfan exposure. In the present study, we elucidated the dose-exposure-response relationship of treosulfan and its metabolite S, S-EBDM. We identified higher treosulfan exposure to be associated with better HCT outcomes. Specifically, lower treosulfan exposure was associated with graft rejection. Also, low treosulfan exposure showed a trend of significance to increased mortality. Though S, S-EBDM exposure alone did not influence HCT outcomes, higher S, S-EBDM to treosulfan exposure was associated with an increased incidence of SOS. We then arrived at a therapeutic cutoff of 1,660 mg•hour/L for better TFS and OS.

Unlike busulfan, where underexposure and overexposure results in poor HCT outcomes, a therapeutic window could not be assigned for treosulfan. However, we can propose that higher treosulfan exposure (>1,660 mg•hour/L) benefits patients with TM undergoing HCT. A recent study by Chiesa *et al*.^37^ demonstrated that target cumulative exposure of 4,800 mg•h/L for treosulfan provided the highest likelihood of survival and sustained engraftment in 87 children, the majority with primary immunodeficiency. However, a more recent study by Van der Stoep *et al*. failed to observe any association between treosulfan exposure and early/late HCT outcomes in 110 pediatric patients with nonuniform malignant disorders. Although higher treosulfan exposure increases the risk of skin toxicity, the authors claim that there is no benefit of treosulfan TDM as it is not limiting the success of HCT.^18^ More recently, Sebastian *et al*. performed a multicenter dosing simulation PK study in 53 children with malignant and nonmalignant hematological disorders and demonstrated that model-based dosing was more accurate than BSA-based conventional dosing, especially for young children.^38^

We propose a therapeutic cutoff (>1,660 mg•hour/L) for patients with TM undergoing HCT with treosulfan-based conditioning. We also suggest that the proposed therapeutic cutoff should be cautiously evaluated in other diagnoses before implementation as clinical outcomes differ among the underlying diagnosis. Strikingly, we also observed that in the high exposure group (>2,400 mg•hour/L), there were no graft rejections, lesser incidence of RRTs (one patient developed SOS), and one death (a patient died due to dengue) (Figure S4). Our results indicate that TDM-based dose escalation would benefit patients, especially in the underexposure group, minimizing graft rejection and early TRM without resulting in treatment-related toxicities as reported previously.^18,39^

Since previous studies showed^7^ that treosulfan undergoes spontaneous nonenzymatic conversion to its metabolite, treosulfan pharmacogenetics has not been explored so far. Functional polymorphisms in the promoter region of the *GSTA1* influence enzyme activity affecting busulfan PK, thereby influencing HCT outcomes. Patients with GSTA1*B are poor metabolizers of busulfan, thereby exhibiting high systemic busulfan exposure, leading to organ toxicities and adverse HCT outcomes.^40–43^ Romański *et al*.^44^ performed a kinetic analysis to examine treosulfan–GSH conjugation *in vitro* and showed that treosulfan does not undergo spontaneous or GST-mediated conjugation with GSH. We screened for GSTA1*B haplotype, which was shown to explain variability in busulfan PK in the present study, and assessed the effect of the genotype on treosulfan/S, S-EBDM exposure. We observed that the patients carrying variant genotypes for GSTA1*B polymorphism had increased S, S-EBDM exposure. It is possible that patients with a variant genotype for GSTA1*B polymorphism have reduced capacity to clear/detoxify the epoxy metabolite resulting in increased S, S-EBDM exposure.

We also performed an exploratory analysis to study treosulfan metabolism using the DMET array. Interestingly, we identified a novel marker, 3′UTR polymorphism, in the *NQO1* gene (rs10517) associated with treosulfan PK. Concordant with our previous findings, this polymorphism was associated with decreased treosulfan and S, S-EBDM exposure. It is possible that patients with variant genotypes for *NQO1* rs10517 polymorphism are good metabolizers of treosulfan as they exhibited increased treosulfan and S, S-EBDM clearance. Ours is the first study to explore the role of genetic variants in explaining the interpatient variability in treosulfan/metabolite exposure.

Since phase II detoxification enzymes GSTA1*B and *NQO1* rs10517 polymorphisms explained treosulfan and S, S-EBDM PK variability, we tested whether these SNPs could influence HCT outcomes. We observed that patients with *NQO1* and GSTA1*B variant genotypes had significantly inferior 1-year OS and TFS. To validate our findings, we further evaluated the impact of these polymorphisms in a large extended retrospective cohort of 314 patients with TM. Strikingly, only the GSTA1*B variant genotype significantly impacted poor survival post-HCT.^45^ Previous studies have demonstrated that GSTA1*B polymorphism is a predictive biomarker for survival and treatment-related toxicities in patients receiving busulfan-containing conditioning regimens.^42,46^ GSTA1*B polymorphism may affect treosulfan metabolism, increasing S, S-EBDM exposure and causing early toxicities and GVHD, resulting in inferior survival. We demonstrated that GSTA1*B could be a plausible prognostic biomarker in HCT with treosulfan-based conditioning.

Limitations of the present study include its nonrandomized nature and lack of validation cohort to confirm the proposed therapeutic cutoff. Our study suggests that lower treosulfan exposure predicts rejection and survival after HCT, and it is possible to make targeted dose adjustments to achieve optimum treosulfan exposure in patients with β-thalassemia major. A prospective trial is warranted to validate the present findings.

## Supplementary Material

Supplementary material

## Figures and Tables

**Table 1:** Patient demographics

Patient characteristics	*N*/Median (range)	%
Age (y)	8 (2–21)	NA
Sex (male/female)	42/35	55/45
Body weight (kg)	21 (11–67)	NA
Lucarelli classification		
Class I/II/III	02/15/60	03/19/78
Vellore risk classification		
Class III high risk/low risk	19/41	25/53
Stem cell source		
Peripheral blood (PBSC)	77	100
HLA Disparity		
Full match	75	97
Not full match	02	03
Donor source		
Matched sibling donor	68	88
Matched unrelated donor	09	12
Cell dose (CD34^+^ × 10^6^/kg)	10 (03–19)	NA
Serum ferritin (ng/mL)	2,654 (100–9,812)	NA
Serum creatinine (mg/dL)	0.26 (0.13–0.74)	NA
Treosulfan formulation		
Generic	45	58
Innovator	32	42

Categorical variables are displayed as *n* (%). Continuous variables are expressed as median (median, range). NA, not applicable; PBSC, peripheral blood stem cell.

**Table 2:** Frequency of genetic variants with potential association with treosulfan and metabolite S, S-EBDM AUC

Genetic polymorphisms	Homozygous reference *N* (%)	Heterozygous *N* (%)	Homozygous *N* (%)	MAF	Indian MAF^a^
GSTA1*B haplotype	36 (47%)	35 (45%)	06 (8%)	0.32	0.32
*NQO1* (rs10517)	51 (66%)	26 (34%)	–	0.17	0.17
*GSTA4* (rs7496)	55 (71%)	20 (26%)	02 (3%)	0.16	0.19
*GSTZ1* (rs1046428)	53 (69%)	20 (26%)	04 (5%)	0.18	0.19
*CES2* (rs4783745)	38 (50%)	33 (43%)	05 (7%)	0.28	0.23

AUC, area under the concentration-time curve; CES2, Carboxylesterase 2; GST, Glutathione S-transferase; NQO1, NAD(P)H Quinone Dehydrogenase 1; —, not applicable.

a MAF, minor allele frequencies obtained from IndiGenomes database.

**Table 3:** Univariate and multivariate Cox regression analysis for predictors of 1-year TFS

		Univariate				Multivariate	
Variable (s)	Risk	95% CI	*P* value		Risk	95% CI	*P* value
Higher age	1.09	0.97–1.23	0.13		–	–	–
Vellore risk classification	1.00	1.00–1.00	0.94		–	–	–
CD34^+^ cell dose	1.13	0.93–1.37	0.21		–	–	–
Donor source	2.16	0.60–7.75	0.23		–	–	–
Pre-HCT serum ferritin levels	**1.00**	**1.00–1.00**	**0.06**		–	–	–
GSTA*B variant genotype	**3.65**	**1.02–13.10**	**0.05**		**3.75**	**1.04–13.47**	**0.04**
*NQO1* rs10517 variant genotype	**2.98**	**1.03–8.61**	**0.04**		**–**	**–**	**–**
Treosulfan AUC <1,660 mg•hour/L	**0.32**	**0.11–0.92**	**0.04**		**3.23**	**1.12–9.34**	**0.03**

1-year TFS was modeled stepwise using Cox proportional hazards using SPSS. Bold values represent *P*-value less than 0.05. AUC, area under the curve; CI, confidence interval; GST, glutathione S-transferase; HCT, hematopoietic stem cell transplantation; HR, hazard ratio; NQO1, NAD(P) H dehydrogenase 1; TFS, thalassemia-free survival; —, variables not taken forward for multivariate analysis.

## Data Availability

The data supporting this study’s findings are available from the corresponding author upon reasonable request.

## Study Highlights

### What is the Current Knowledge on the Topic?

Treosulfan-based conditioning regimen is increasingly used in hematopoietic stem cell transplantation (HCT) owing to a tolerable toxicity profile but excellent myeloablative properties. Few studies have evaluated the impact of treosulfan exposure on HCT outcomes suggesting a moderate exposure-toxicity relationship, but a relationship with survival is not consistent across observations.

### What Question did this Study Address?

Pharmacokinetics, pharmacogenetics, and pharmaco-dynamics of treosulfan and its metabolite S, S-EBDM were evaluated in patients with β-thalassemia major. Dose-exposure-response relationship of treosulfan and S, S-EBDM were evaluated to identify whether therapeutic drug monitoring (TDM) and dose adjustment of treosulfan is feasible.

### What does this Study Add to our Knowledge?

Treosulfan exposure is a strong predictor for thalassemia-free survival; suboptimal exposure (<1,660 mg•hour/L) leads to increased graft rejection and decreased survival. Treosulfan, not its active metabolite S, S-EBDM influences HCT outcomes, especially survival post-HCT. GSTA1*B polymorphism could be a prognostic biomarker for HCT with a treosulfan-based regimen.

### How Might this Change Clinical Pharmacology or Translational Science?

TDM-based treosulfan dosing could help in optimizing HCT outcomes.
