# Supplementary material for: Treosulfan Exposure Predicts Thalassemia-Free Survival in Patients with Beta Thalassemia Major Undergoing Allogeneic Hematopoietic Cell Transplantation
Source: Clin Pharmacol Ther. Author manuscript; Available in PMC 2024 Mar 30. (PMC7615782; doi:10.1002/cpt.3078)
Supplement: Supplementary material [file EMS194925-supplement-Supplementary_material.docx]

**SUPPLEMENTARY MATERIAL**

**Treosulfan Exposure Predicts Thalassemia-free Survival In Patients With Beta Thalassemia Major (TM) Undergoing Allogeneic Hematopoietic Cell Transplantation**

**Aswin Anand Pai^1,2^**, Ezhilpavai Mohanan^1^, John C Panetta^3^, Uday P. Kulkarni^1^, Stallon Illangeswaran RS^1^, Balaji Balakrishnan^1^, Agila Jayaraman^1^, Eunice S Edison^1^, Kavitha ML^1^, Anup J. Devasia^1^, Fouzia NA^1^, Anu Korula^1^, Aby Abraham^1^, Biju George^1^, Alok Srivastava^1^, Vikram Mathews^1^, Joseph F Standing^4, 5^, Poonkuzhali Balasubramanian^1^

*^1^Department of Haematology, Christian Medical College, Vellore, India*

*^2^Sree Chitra Tirunal Institute for Medical Sciences and Technology, Thiruvananthapuram, Kerala, India*

*^3^Department of Pharmaceutical Sciences, St Jude Children’s Research Hospital, Memphis, TN, USA*

*^4^Infection, Immunity and Inflammation, Great Ormond Street Institute of Child Health, University College London, London, UK.*

*^5^Department of Pharmacy, Great Ormond Street Hospital for Children, NHS Foundation Trust, London, UK.*

**Correspondence:**

**Poonkuzhali Balasubramanian, Ph.D.**

Professor

Department of Haematology

Christian Medical College

Ranipet Campus 632517

Tel No: +91-4172-24576

E-mail: bpoonkuzhali@cmcvellore.ac.in

ORCID ID: 0000-0002-9443-439X

**Table of Contents**

**Supplementary Figures**

**Figure S1: Representative Chromatogram showing Treo, metabolite S, S-EBDM, and Internal standard (4′-Aminoacetophenone, AAP)……………………………………………………………………………3**

**Figure S2: Basic goodness-of-fit plots of the final model ………..……………………………...…………3**

**Figure S3: Impact of *NQO1* polymorphism on 1-year OS and TFS……………………………...…………4**

**Figure S4: Impact of *GSTA1*B* polymorphism on 1-year OS and TFS…………………………..………..4**

**Figure S5: PD model fit of the quadratic expression describing the change in probability of success (defined by 1-year TFS) with increasing Treo AUC………………….……………………………….5**

**Supplementary Tables**

**Table S1: Mass transitions and optimized MS/MS parameters for quantification of Treo and S, S-EBDM with 4’-AAP as Internal standard…………………………………………………………….…………5**

**Table S2: Representative table showing MS validation parameters – Inter-day Accuracy and Precision………………………………………………………………………………………………………………………..…6**

**Table S3: Cox proportional hazards model for 1-year Mortality and Graft Rejection…………….6**

**Table S4: Comparison of Treo PK with previous reports………………………………………………………7**

**R Model codes for PK-PD analysis……………………………………………………………………………10**

**Figure S1: Representative Chromatogram showing Treo, metabolite S, S-EBDM, and Internal standard (4′-Aminoacetophenone, AAP)**

**
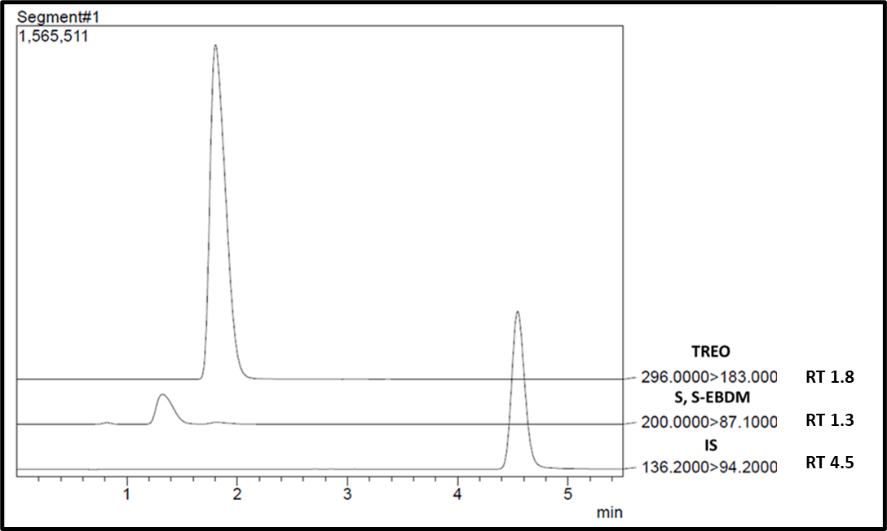
**

Representative chromatograms were obtained while analyzing the Mid QC sample of Treo and S, S-EBDM spiked in blank plasma.

**Fig S2: Basic goodness-of-fit plots of the final model**


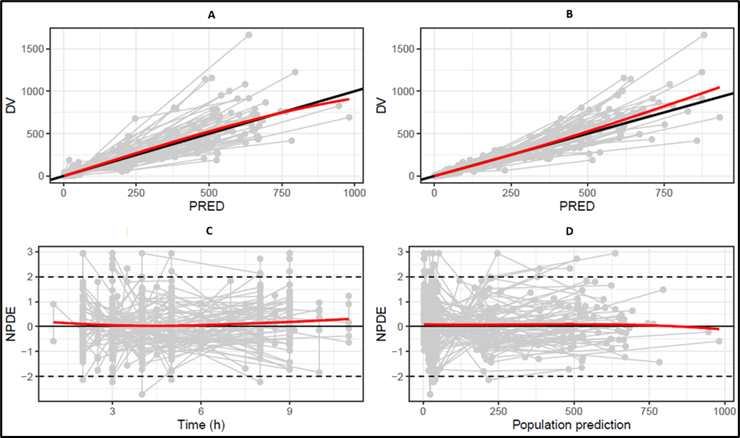


(A) observations versus population predictions; (B) observations versus individual predictions; (C) conditional weighted residuals versus time; (D) conditional weighted residuals versus population predictions. Observed versus population predicted concentrations (IPRED, left) and observed versus individual predicted concentrations (PRED, right) for the final model. The solid black line represents the line of identity. Conditional weighted residuals (CWRES) versus time on the lower left and PRED on the lower right. Points are individual data. Red dashed lines represent regression. Black dashed lines represent |CWRES|

**Figure S3 A: Impact of *NQO1* polymorphism on 1-year OS and TFS**

**
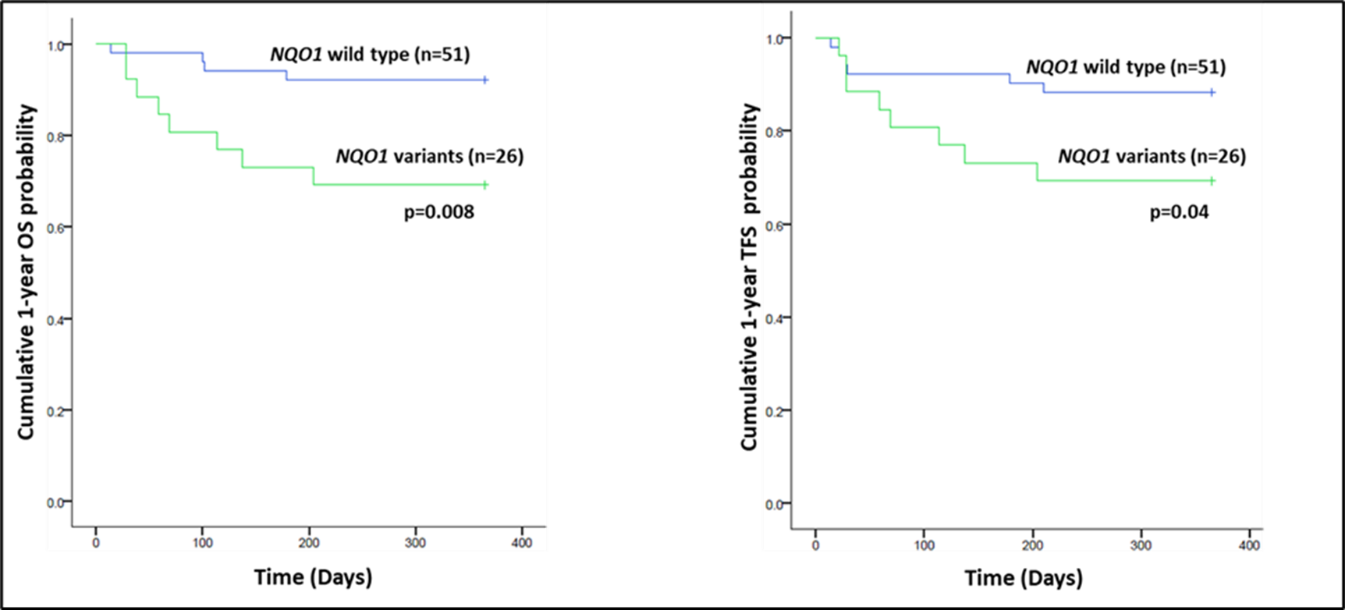
**

Kaplan-Meier survival curves showing associations between *NQO1* 3’UTR variant (rs10517) genotype with 1-year OS (left) and TFS (right).

**Figure S3 B: Impact of *GSTA1*B* polymorphism on 1-year OS and TFS**

**
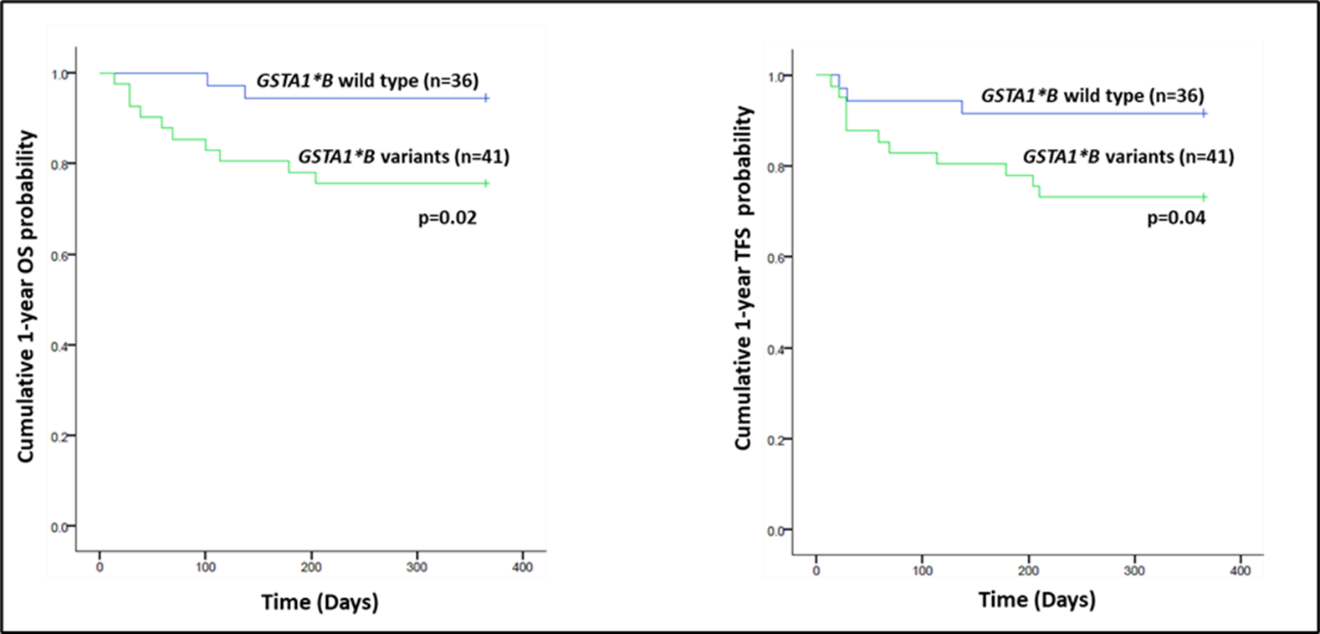
**

Kaplan-Meier survival curves showing associations between *GSTA1*B* promoter polymorphism variant genotype with 1-year OS (left) and TFS (right).

**Figure S4: PD model fit of the quadratic expression describing the change in probability of success (defined by 1-year TFS) with increasing Treo AUC.**

**
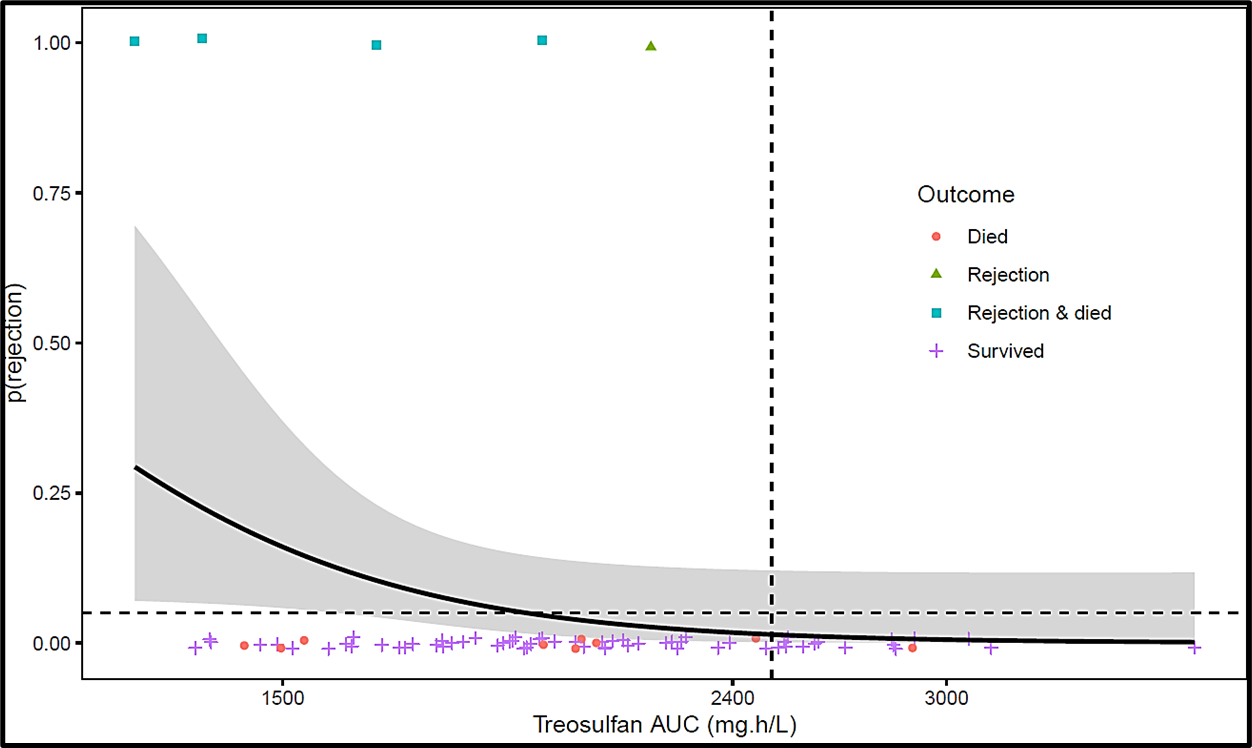
**

The black line and associated shaded area are the model fit and 95% confidence interval, plus are AUC for patients with successful outcomes; squares are for patients who had rejected their grafts and died subsequently, triangles are for patients who had graft rejection but alive, and circles are patients who died due to other causes. The vertical dashed line gives AUC at which the probability of success is maximized.

**Table S1: Mass transitions and optimized MS/MS parameters for quantification of Treo and S, S-EBDM with 4’-AAP as Internal standard**

**
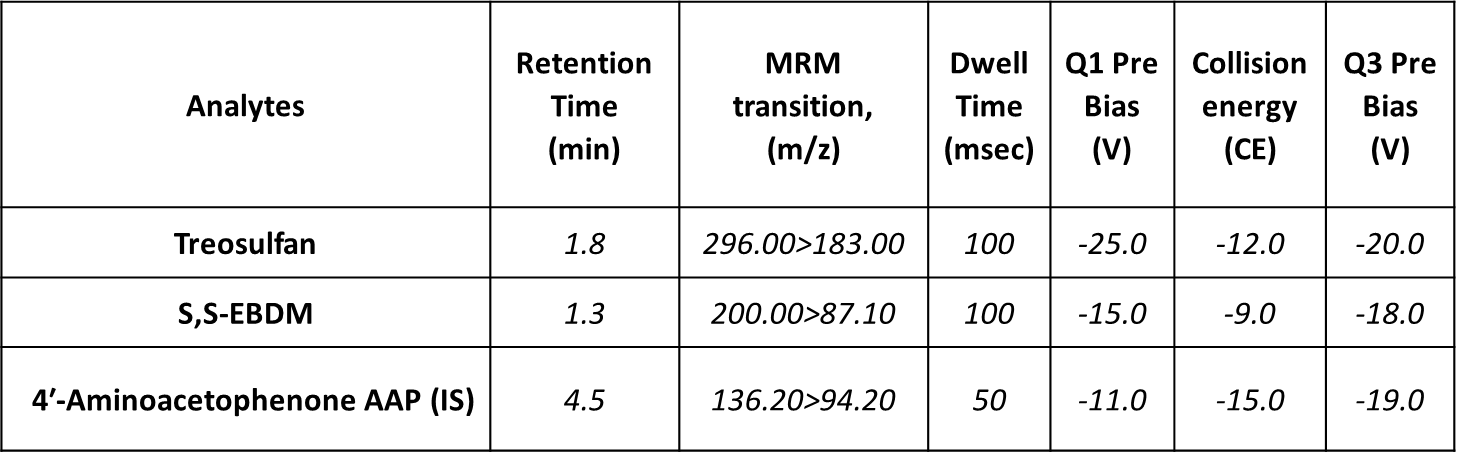
**

Optimized MRM transitions used for quantification of Treo and S, S-EBDM by an LC-MS/MS-based assay.

MRM, Multiple reaction monitoring; Q, Quadrupole mass filter.

**Table S2: Representative table showing MS validation parameters – Inter-day Accuracy and Precision**

**
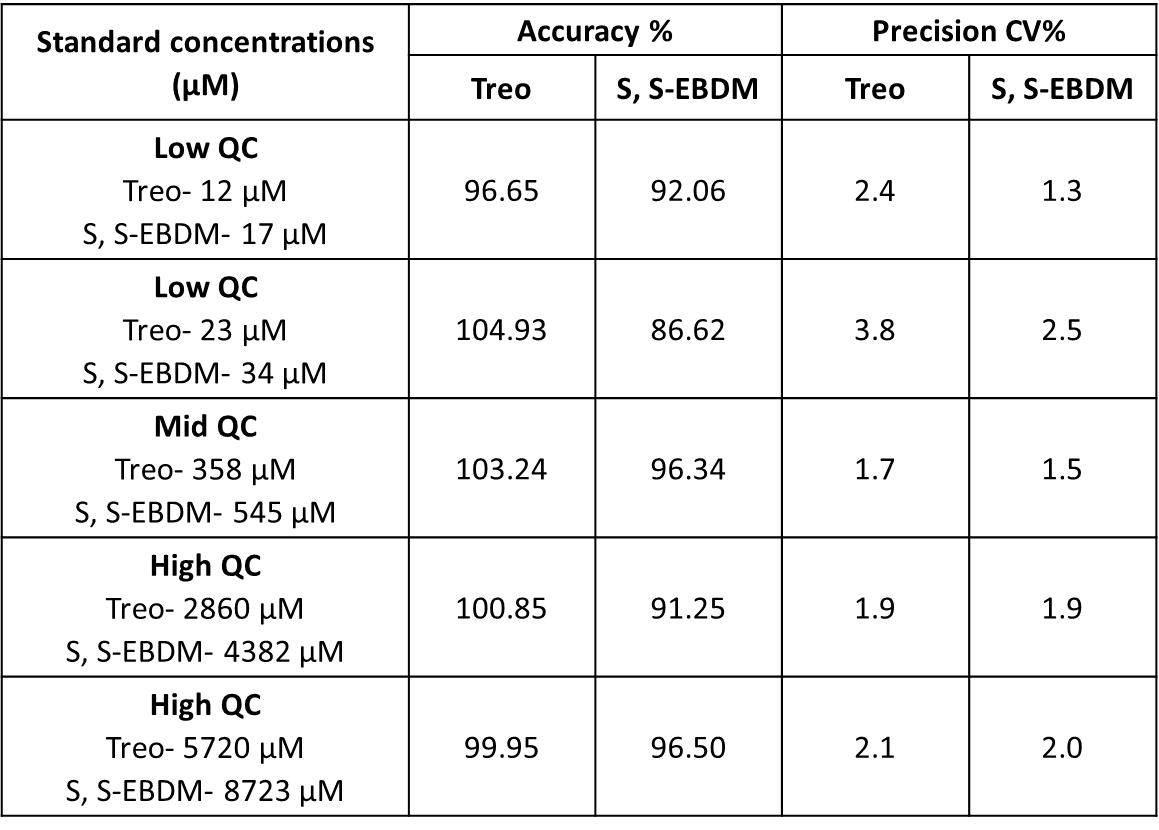
**

Inter-day precision and accuracy were calculated based on individual experiments on five days. The inter-day CV% was <5%.

CV, coefficient of variation.

**Table S3: Cox proportional hazards model for 1-year Mortality and Graft Rejection**

**
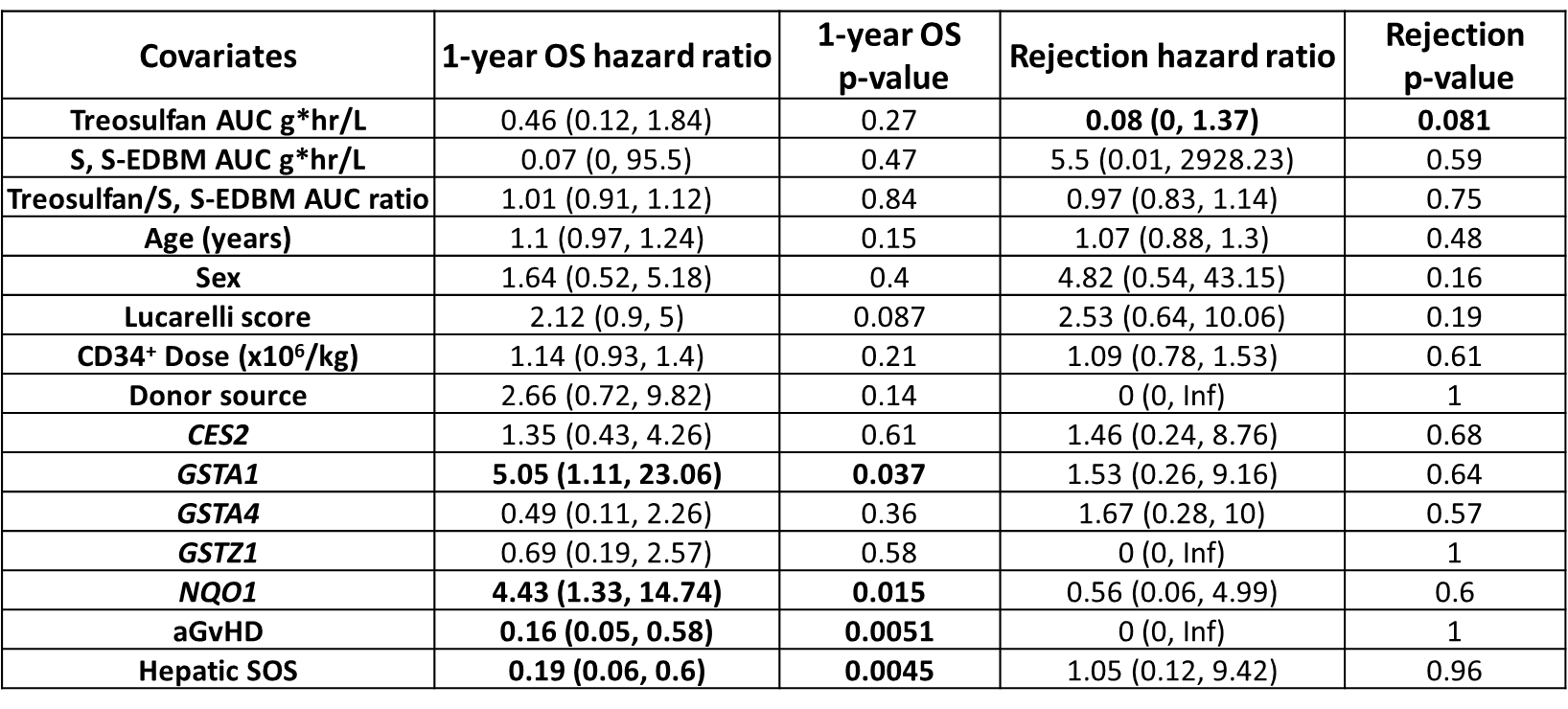
**

1-year Mortality and Graft rejection were modeled stepwise using Cox proportional hazards using R statistics.

AUC, Area under the curve; GST, Glutathione S-transferase; *NQO1*, NAD(P)H dehydrogenase 1; CES2, Carboxylesterase 2; SOS, Sinusoidal Obstruction Syndrome.

**Table S4: Comparison of Treo PK with previous reports**

Treo PK parameters were comparable to the previous studies.

HM- Haematological malignancies, IEI- Inborn errors of metabolism, GD- Genetic diseases, HBP- Haemoglobinopathies, BMF- Bone marrow failure, IBD- Inflammatory Bowel Disease, JMML- Juvenile myelomonocytic leukemia, β-TM- beta thalassemia major, Treo- Treosulfan, Flu- Fludarabine, ThioT- Thiotepa.

| **S.No** | **Diagnosis** | **N** | **Age**  **Median (Range)** | **Conditioning**  **Regimen** | **Treo Dose** | **Day 1 Treo**  **Exposure (AUC)**  **mg*h/L** | **Day 1 Treo Clearance**  **L/h** | **Significant Findings** | **Refs** |
| --- | --- | --- | --- | --- | --- | --- | --- | --- | --- |
| **1** | HM- 15 (28.3%)  IEI- 32 (60.4%)  GD (11.3%) | 53 | 3.5  (0.9 – 12) | Treo/Flu/ThioT- 26 (49%)  Treo/Flu- 22 (41.5%)  Treo/Flu/Mel- 4 (7.5%)  Treo/Mel- 1 (1.9%) | 30 g/m^2^- 6 (11.3%)  36 g/m^2^- 11 (20.8%)  42 g/m^2^- 36 (67.9%) | HM- 1649  (1419 – 1889)  IEI- 1648  (1457 – 1896)  GD- 1634  (1407 – 1876) | 16.4  L/h/70 kg | Model-informed recommended for patients <2 years.  No outcomes were evaluated. | 38 |
| **2** | IEI- 38 (35%)  HBP- 55 (50%)  BMF- 17 (15%) | 110 | 5.2  (0.2-18.8) | Treo/Flu- 37 (32%)  Treo/Flu/ThioT- 77 (68%) | 30 g/m^2^- 18 (16%)  42 g/m^2^- 92 (84%) | 30 g/m^2^-1776  (IQR, 1129 to 1977)  42 g/m^2^- 1562  (IQR, 1140 to 1860) | - | Mucositis was associated with high Treo AUC; No associations with other clinical outcomes including survival or toxicities. | 18 |
| **3** | IEI- 79 (91%)  IBD- 5 (6%)  JMML- 2 (2%)  IEM- 1 (1%) | 87 | 1.6  (0.2-16.7) | Treo/Flu (100%) | 30 g/m^2^- 4 (5%)  36 g/m^2^- 23 (26%)  42 g/m^2^- 60 (69%) | 30 g/m^2^-4,521  (4,352–4,740),  36 g/m^2^- 5,204  (2,321–9,023),  42 g/m^2^- 4,590  (2,880–14,647) | 17.31  L/h/70 kg | A cumulative Treo AUC of 4,800 mg*h/L maximized the  probability of success  (> 20% engraftment & no mortality) at 82%. | 17 |
| **4** | HBP- 31(40%)  HM- 12 (16%)  IEI- 22 (29%)  BMF- 11 (14%)  Other- 1 (1%) | 77 | 4.8  (0.2-18.3) | Treo/Flu- 25 (36%)  Treo/Flu/ThioT- 52 (64%) | 30 g/m^2^- 12 (16%)  42 g/m^2^- 65 (84%) | 30 g/m^2^- 1561  (511–3250)  42 g/m^2^- 1744  (732-3,544) | 6.98  L/h/20 kg | High IIV in Treo PK and high Treo AUC was associated with early toxicities including skin and mucositis. | 16 |
| **5** | β-TM | 87 | 9.0  (1.5-25) | Treo/Flu/ThioT- 77 (100%) | 42 g/m^2^- 100% | 1,326  (126–4,484) | 11.2  L/h/m^2^ | High IIC in Treo PK; no clearcut associations with HCT outcomes | 21 |
| **7** | β-TM | 77 | 8.0  (2.0-21) | Treo/Flu/ThioT- 77 (100%) | 42 g/m^2^- 100% | 1993  (1286-3886) | 14.02  L/h/70Kg | Low Treo exposure predicts graft rejection, Treo cut-off>1660 predicts better 1-year TFS | *Present study* |

**R model code for PK model**

library(nlmixr2)

### Treosulfan (parent)- S,S, EBDM (metabolite) combined error model with allometric scaling ###

treo_par_metab_allo <- function() {

ini({

tcl <- log(6) # clearance

tv <- log(20) # volume

tclm <- log(30) # metabolite clearance

tvm <- log(100) # metabolite volume

eta.cl + eta.v + eta.clm + eta.vm ~ c(0.1,

0.01, 0.1,

0.01, 0.01, 0.1,

0.01, 0.01, 0.01, 0.1)

add.err <- 1 # residual variability

prop.err <- 0.1 # residual variability

add.err.m <- 0.1 # residual variability

prop.err.m <- 0.1 # residual variability

})

model({

cl <- exp(tcl + eta.cl + (0.75 * LOGWT70)) # individual value of cl

v <- exp(tv + eta.v + LOGWT70) # individual value of v

clm <- exp(tclm + eta.clm + (0.75 * LOGWT70)) # individual value of cl

vm <- exp(tvm + eta.vm + LOGWT70) # individual value of v

d/dt(A_c) = -cl / v * A_c

d/dt(A_m) = cl / v * A_c - clm / vm * A_m

# output auc

auc <- DOSEMG / cl

aucm <- DOSEMG / clm

ct <- A_c / v

ct ~ add(add.err) + prop(prop.err) # define error model

cm <- A_m / vm

cm ~ add(add.err.m) + prop(prop.err.m) # define error model

})

}

# Check the model

nlmixr(treo_par_metab_allo)

# Fit the model

fittreo_par_metab_allo_S <-

nlmixr(

treo_par_metab_allo(), #the model definition

pk.treo.1, #the data set

est = "saem", #the estimation algorithm (SAEM)

#the SAEM minimisation options:

saemControl(nBurn = 200, #200 SAEM burn-in iterations (the default)

nEm = 300, #300 EM iterations (the default)

print = 50), #print every 50th iteration

tableControl(cwres = TRUE,npde=TRUE) #calculates NONMEM-style conditional weighted residuals and npde for diagnostics

)

fittreo_par_metab_allo_S
